# A novel predictive model for phthisis bulbi following facial hyaluronic acid cosmetic injection

**DOI:** 10.1186/s12886-023-02992-4

**Published:** 2023-05-31

**Authors:** Shancheng Si, Wei Su, Lei Wang, Yicong Ji, Anming Chen, Yuntao Hu

**Affiliations:** 1 Eye Center, Beijing Tsinghua Changgung Hospital, School of Clinical Medicine, Tsinghua University, Beijing, 102218 China; 2 Department of Neurosurgery, Beijing Tsinghua Changgung Hospital, School of Clinical Medicine, Tsinghua University, Beijing, 102218 China

**Keywords:** Anterior segment ischemia, Ciliary hyposecretion, Cosmetic filler injection, Hyaluronic acid, Phthisis bulbi, Hypotony, Anterior segment circulation, New injury severity scale

## Abstract

**Purpose:**

To observe long-term prognosis of anterior segment ischemia (ASI) following hyaluronic acid (HA) injection, propose a severity grading system for ASI and a predictive model for phthisis bulbi (PB) based on long-term secretion dysfunction of ciliary process.

**Methods:**

This is a retrospective case–control study. All enrolled 20 patients were divided into two groups and followed for at least 6 months to observe the formation and transformation characteristics of ASI and long-term prognosis based on the degrees of ciliary function damage.

**Results:**

The severity of ASI following HA injection could be subdivided into 4 grades according to the degrees of ciliary function damage, comprising ASI grades 0, 1, 2 and 3. In 20 patients, ophthalmoplegia at 1-month follow-up, ASI within 1 month, ASI at 1-month follow-up, hypotony within 6 months were all significantly more common in study group than in control group (60% vs. 0%, *P* = 0.011; 100% vs. 20%, *P* = 0.001; 100% vs. 0%, *P* < 0.001; 80% vs. 0%, *P* = 0.001, respectively). Sensitivity, specificity and the area under the receiver operating characteristic curve (AUC) for predicting subsequent PB at 2-year follow-up through the co-occurrence of ophthalmoplegia at 1-month follow-up and hypotony within 6 months was 100%, 100% and 1.00, respectively.

**Conclusions:**

The new grading system for ASI and novel predictive model for PB we proposed could predict the long-term prognosis and probability of subsequent PB due to ASI following HA injection through several dynamic assessments within 6 months.

**Level of Evidence:**

Level IV, observational prognostic study.

## Introduction

Facial cosmetic fillers can cause a variety of ocular complications, which is gradually attracting the attention of doctors and patients. These complications can range from mild vision loss or visual field loss [1] to blindness, anterior segment ischemia (ASI) or cerebral infarction [2] However, most of the literature only describes the damage of patients in the acute phase, and there are few long-term observations of more than 6 months [1–3]. Our previous research has concerned the therapeutic effect of intraarterial thrombolysis on ophthalmic artery occlusion (OAO) in the acute phase [4].

To further investigate its long-term complications of ASI following cosmetic filler injection, we conducted a 2-year follow-up of the previous 22 patients [5] who first visited our hospital between June 2017 and February 2021. It was found that 10 out of the 22 patients underwent various degrees of long-term ciliary process dysfunction after facial cosmetic filler injection. In this article, we systematically summarize these long-term ciliary process dysfunctions secondary to ASI following cosmetic filler injection and possible pathophysiological mechanisms.

## Methods

### Subjects

Long-term ciliary process dysfunction secondary to ASI following cosmetic filler injection occurred in 10 out of 22 patients [5] evaluated on Department of Ophthalmology at Beijing Tsinghua Changgung Hospital between June 2017 and February 2021. The long-term ciliary process dysfunction secondary to ASI over 6 months in all 10 patients, including phthisis bulbi, hypotony and ciliary hyposecretion, were retrospectively reviewed and then compared with those without such complications. Institutional Review Board approval was obtained from Beijing Tsinghua Changgung Hospital, and this study adhered to the tenets of the Declaration of Helsinki. An informed consent form was signed by every enrolled patient before the start of standardized form recording and detailed ocular/orbital examinations.

### Grouping and follow-up criteria

A total of 22 cases have been described in detail in our previous study [5]. Among them, two patients receiving facial cosmetic injection of autologous fat instead of HA were excluded. And, 10 patients suffering from one of the above 3 long-term ciliary process dysfunction were enrolled in “the study group”. As a result, the remaining 10 cases were enrolled in “the control group”. All enrolled patients in “the study group” and “the control group” were followed up for at least 6 months and up to 24 months, with the follow-up time point in Month 1, 6, 12, 24 after injury.

### Ocular/orbital examinations

All enrolled subjects were subjected to a series of ocular/orbital examinations listed as follows: (1) intraocular pressure (IOP) through non-contact tonometer; (2) slit-lamp microscopy; (3) fundus photography; (4) axial length examination by Lenstar (Haag- Streit AG). For patients with predisposition to phthisis bulbi (PB) or orbital involvement, magnetic resonance imaging (MRI) or computed tomography was added for further evaluation. Since, 9 of 10 enrolled in “the study group” had the vision of no light perception (NLP).

### Definitions of terms in this study

#### Phthisis Bulbi (PB)

**Fig. 1:**
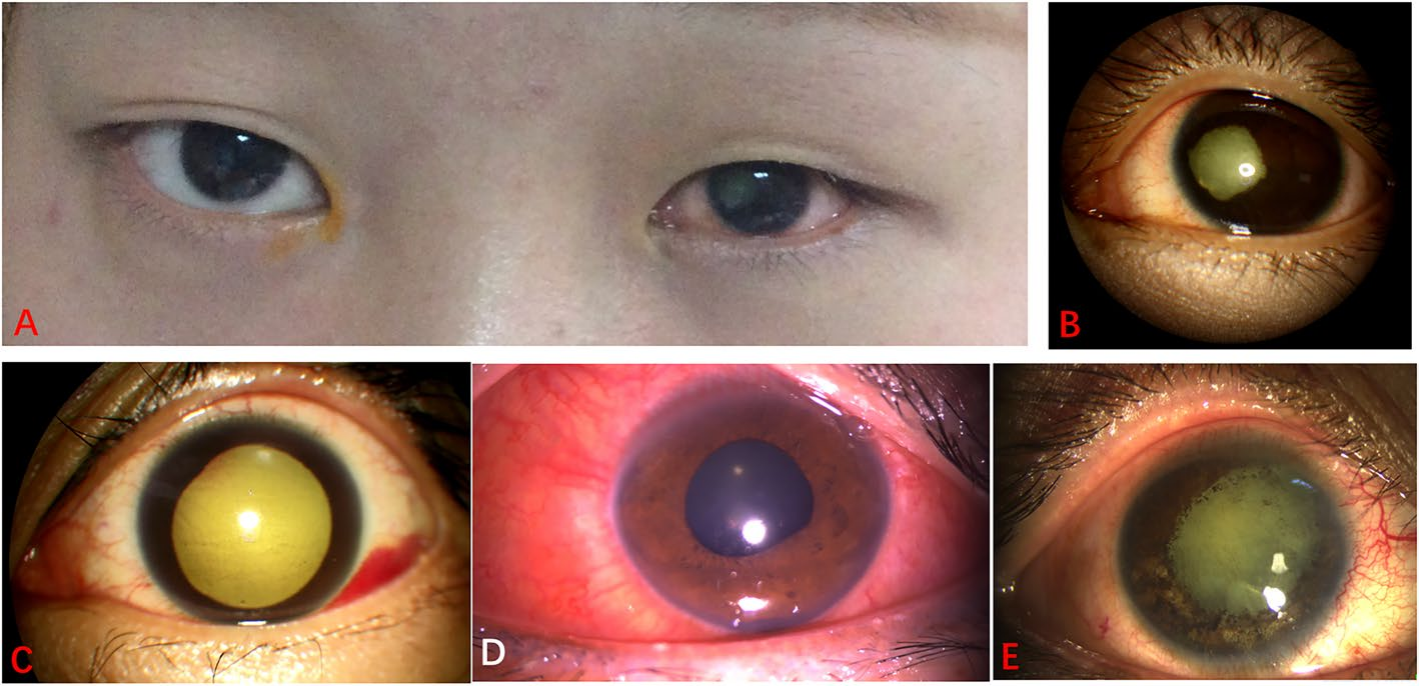
Typical imaging features of 4 grades of ASI following facial cosmetic filler injection. **A**, **B** *Grade 3*. Photography of binocular external aspect and left anterior segment of case No. 6 diagnosed with PB showing ptosis, shrunk globe, enophthalmos, mild conjunctival hyperemia, 360-degree circumferential iris atrophy, pupil shift temporally and cataract, of the left eye, taken in approximately the 6th month after ASI following facial HA injection. **C** *Grade 2*. Left anterior segment photography of case No. 1 diagnosed with hypotony showing subconjunctival hemorrhage, mild corneal edema, Tyndall effect, mydriasis, pupil irregularity, taken on the 7th day after ASI following facial HA injection. **D** *Grade 1*. Left anterior segment photography of case No. 13 diagnosed with CHS showing severe mixed conjunctival hyperemia, mild corneal edema, positive Tyndall effect, mid-dilated and irregular pupil, taken on the 12th day after ASI following facial HA injection. **E** *Grade 2*. Right anterior segment photography of case No. 3 diagnosed with hypotony showing mild conjunctival hyperemia, 360-degree circumferential iris atrophy, mydriasis, pigmentation on lens surface and cataract, taken in approximately the 1.5 months after ASI following facial HA injection, but with subsequent PB at 6-month follow-up. ASI, anterior segment ischemia; HA, hyaluronic acid; CHS, ciliary hyposecretion; PB, phthisis bulbi

The pathological state with IOP of the affected eye usually less than 8 mmHg, shortened corneal diameter, thickened ocular coats, disorganized intraocular contents and globe size 20% or more smaller than before injury [6–9] (Fig. 1A, 1B).

#### Hypotony

The pathological state with IOP of the affected eye usually more than 5 mmHg lower than that of the contralateral healthy eye, less than 8 mmHg, with the Tyndall effect caused by disruption of the blood-ocular barrier, but with globe size of the affected eye not 20% smaller than before injury [6–9] (Fig. 1C).

#### Ciliary hyposecretion (CHS)

The pathological state with IOP of the affected eye more than 5 mmHg lower than that of the contralateral healthy eye due to decreased ability of ciliary process to secrete aqueous humor caused by various reasons, but with or without the Tyndall effect caused by disruption of the blood-ocular barrier [10] (Fig. 1D).

#### Anterior segment ischemia (ASI)

The syndrome secondary to hypoperfusion of the anterior segment circulation (ASC) caused by various reasons, with clinical characteristics of segmental or circumferential atrophy of the iris, pupil irregularity, uveitis, corneal edema, CHS, hypotony, cataract, even PB [10–14]. Some manifestations are usually not associated with long-term ciliary process dysfunction, such as segmental iris atrophy, pupil irregularity, uveitis, which we call *mild ASI (mASI).* It should be noted that ophthalmoplegia occurring within 24 h after primary hypoperfusion (or 360°circumferential iris atrophy at 6-month follow-up) combined with hypotony predicts very high likelihood of PB, which we call *severe ASI (sASI)* [10, 15] (Fig. 1E).

#### The new injury severity scale (NISS) for ocular complications following facial cosmetic filler injection [5]

The injury severity of ocular complications following facial cosmetic filler injection based on NISS is divided into 4 grades, comprising NISS grades 1, 2, 3 and 4. The specific contents are as follows: *Grade 1* indicating that BCVA is hand motion or better at the time of injury; *Grade 2* indicating LP at the time of injury or delayed NLP; *Grade 3* indicating immediate NLP at the time of injury; *Grade 4* indicating immediate NLP complicated with stroke at the time of injury.

### Statistical analysis

Through continuous observation for up to 2 years, we first screened for several clinical features that might contribute to **s**ecretion dysfunction of ciliary process (including iris atrophy of 360°circumference, hypotony, ophthalmoplegia, ASI, NISS, NLP, etc.) from the 10 cases in study group and then compared the percentage of these clinical features occurring in study group and control group. Secondly, the clinical features within 6 months with *P* values of < 0.05 on the above hypothesis test were used to predict subsequent PB at 2-year follow-up. Then, we identified 7 scenarios with the duration of at least 1 month, including individual features (namely, scenarios A = iris atrophy of 360°circumference within 6 months, B = hypotony within 6 months, C = ophthalmoplegia at 1-month follow-up, D = ASI at 1-month follow-up) and their combinations (E = “B and C”, F = “B and D”, G = “C and D”), calculated their sensitivity, specificity and predictive accuracy (area under the receiver operating characteristic curve (AUC)) based on the total of 22 cases and compared the AUCs. Finally, we identified the best scenario for predicting subsequent PB.

For quantitative parameters such as “age”, we calculated the mean value and standard deviation. Shapiro–Wilk test was used to test for normal distribution and the student’s t-test for independent samples was used to assess differences in quantitative parameters. For the evaluation of all categorical parameters, Fisher's exact test was applied. *P* values of < 0.05 were considered statistically significant. All statistical analyses were performed using SPSS Version 25 software (IBM Corp., Armonk, NY).

## Results

### Demographic and clinical characteristics of all 10 cases enrolled in “the study group”

**Fig. 2:**
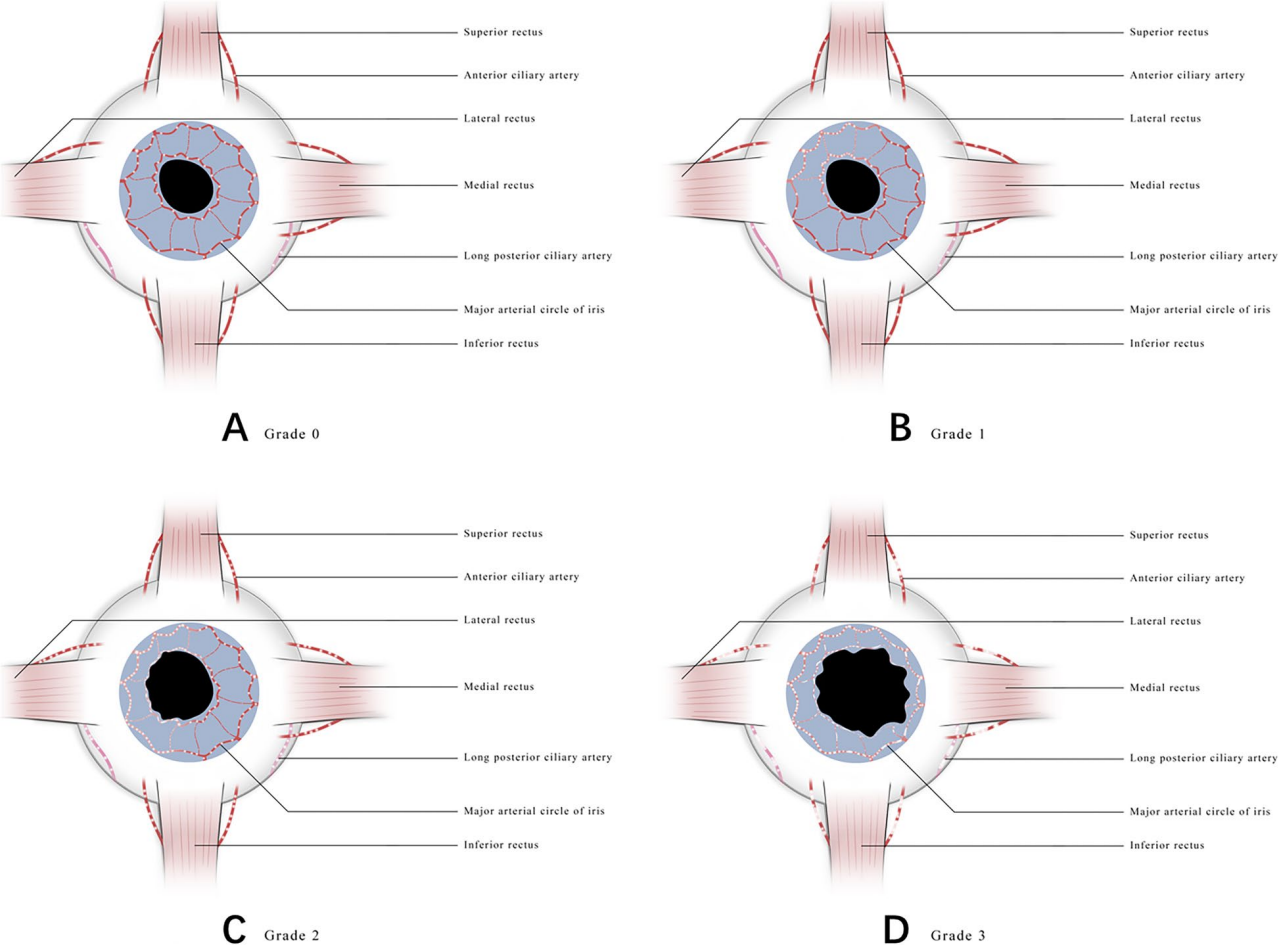
Schematic diagram of 4 grades of ASI in the grading system mentioned in this article. **A** *Grade 0*. When a small amount of HA microspheres blocks the ASC, there is usually no definite ciliary function damage, but may be other manifestations of mASI such as uveitis, pupil irregularity. **B** *Grade 1*. If the HA microspheres are dense in a certain quadrant, it is possible to cause segmental iris atrophy, usually with mild ciliary process dysfunction (CHS), usually with clinical characteristics of mydriasis, uveitis. **C** *Grade 2*. When a large amount of HA microspheres blocks the ASC, it is easy to cause extensive iris atrophy. If no 360-degree circumferential iris atrophy occurs, there may present with moderate ciliary function damage (hypotony), with clinical characteristics of mydriasis, Tyndall effect, severe uveitis, corneal edema, but usually no subsequent PB. **D** *Grade 3*. When a large amount of HA microspheres blocks the ASC, resulting in 360-degree circumferential iris atrophy, there may present with severe ciliary function damage, with clinical characteristics of mydriasis, severe uveitis, corneal edema, cataract and subsequent PB. ASI, anterior segment ischemia; HA, hyaluronic acid; ASC, anterior segment circulation; CHS, ciliary hyposecretion; PB, phthisis bulbi

The average follow-up period was 19.8 months (ranging from 6 to 24 months). After 2 years of follow-ups, the long-term ciliary process dysfunction secondary to ASI following HA injection occurred in 10 eyes (5 right and 5 left eyes) of 10 patients (1 male and 9 females), with the mean age of 33 years (median, 31 years; ranging from 18 to 44 years). Specifically, PB, hypotony, CHS occurred in 5, 3, 2 cases, respectively. On the other hand, from the aspect of structural damage, the iris atrophy of 360°circumference, mydriasis and iris atrophy of 1 quadrant are the three main sequelae of ASI, occurring in 4, 3, 3 cases, respectively. It was interesting to note that all 4 cases (Case No. 2,3,4,18) with 360-degree circumferential iris atrophy developed PB at 1-year follow-up. It can be seen that the secondary impairment of ciliary process function caused by ASI may continue to aggravate during subsequent follow-up. PB, hypotony, CHS could be seen as three different grades of impaired ciliary function, representing grades 3, 2, 1 of the severity of ASI, respectively. The mASI was usually not associated with definitely long-term ciliary function damage, so we divided it into grade 0. Schematic diagram of 4 grades of ASI in the grading system mentioned in this article was depicted in Fig. 2. The demographic and clinical characteristics of all 10 cases enrolled in the study group were listed in Table 1.

**Table 1:** The demographic, clinical characteristics and grade in NISS of all 10 cases enrolled in the study group

Case No	Long-term complications at 2-year follow-up	Gender	Age	Involved eye	Interval from injury to long-term complication onset (months)	IOP at long-term complication onset (mmHg)	Follow-up duration (months)	Grade in NISS	Sequelae of ASI during 2 years of follow-up
1	Hypotony	female	28	OS	1	5	6	4	mydriasis
2	PB	female	27	OD	6	6	24	3	iris atrophy of 360°circumference
3	PB	male	31	OD	6	5.6	24	3	iris atrophy of 360°circumference
4	PB	female	42	OD	6	6.5	24	4	iris atrophy of 360°circumference
6	PB	female	18	OS	6	0	24	3	iris atrophy of 1 quadrant
13	CHS	female	44	OS	1	13.5	12	1	iris atrophy of 1 quadrant
16	CHS	female	30	OD	6	13.8	24	3	mydriasis
18	PB	female	31	OD	12	7.3	24	3	iris atrophy of 360°circumference
20	Hypotony	female	35	OS	6	6.8	24	3	mydriasis
22	Hypotony	female	44	OS	1	7.8	12	3	iris atrophy of 1 quadrant

*CHS* Ciliary hyposecretion, *IOP* Intraocular pressure, *NISS* New injury severity scale, *PB* Phthisis bulbi

### The relationship between NISS and long-term ciliary process dysfunction secondary to ASI

**Fig. 3:**
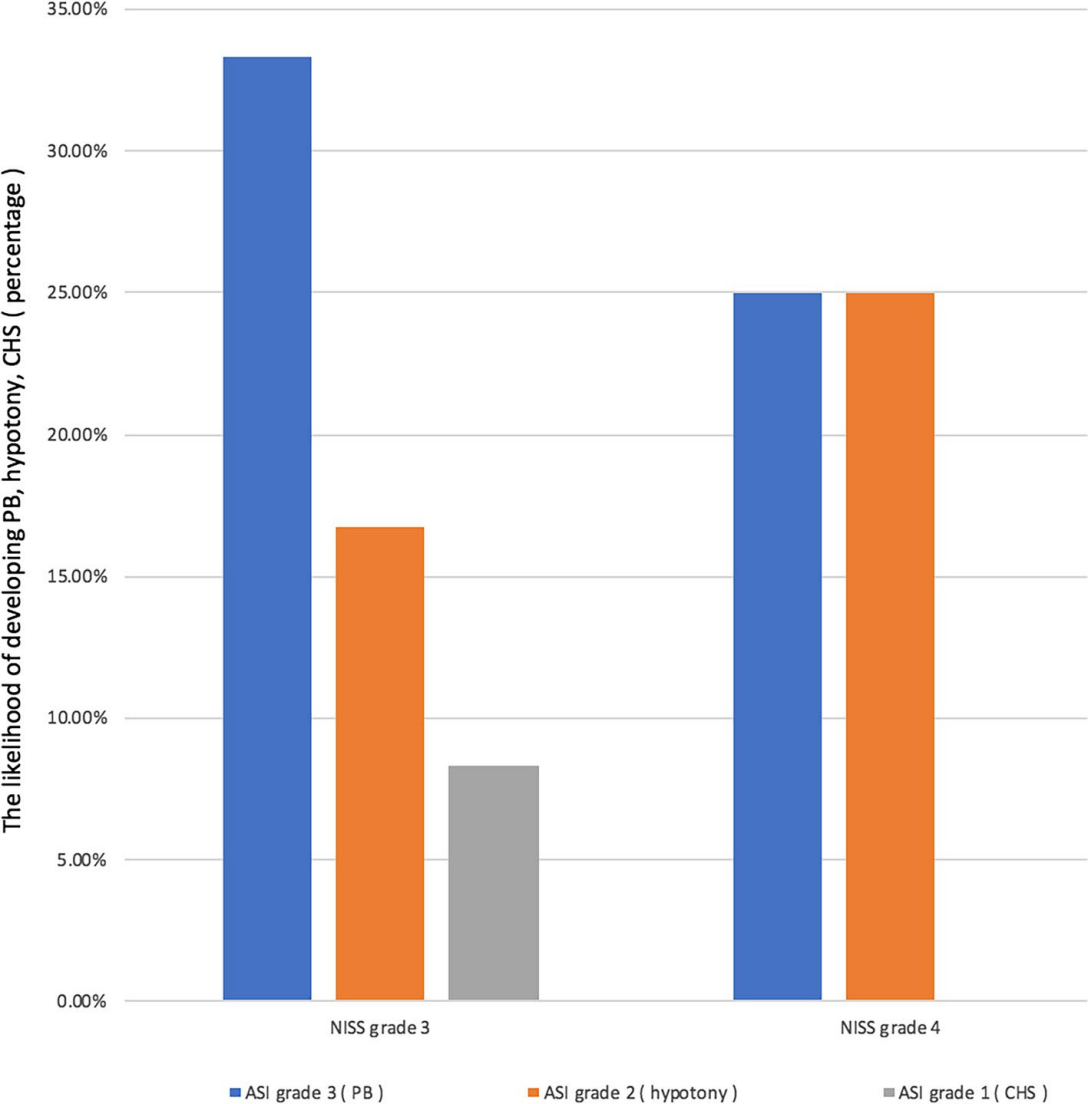
The relationship between NISS and long-term ciliary process dysfunction secondary to ASI. The likelihood of developing PB, hypotony, CHS in NISS grades 3 and 4 were 4/12 (33.3%), 2/12 (16.7%), 1/12 (8.3%) and 1/4 (25.0%), 1/4 (25.0%), 0/4 (0.0%), respectively. ASI, anterior segment ischemia; CHS, ciliary hyposecretion; NISS, new injury severity scale; PB, phthisis bulbi

A total of 12 and 4 cases were classified as grades 3 and 4 in the prior study we proposed NISS. After 2 years of follow-ups, two (PB and hypotony) of the three kinds of long-term ciliary process dysfunctions induced by ASC embolism only appeared in NISS grades 3 and 4, except for CHS, which could occur in NISS grade 1 (Case No. 13). The likelihood of developing PB, hypotony, CHS in NISS grades 3 and 4 were 4/12 (33.3%), 2/12 (16.7%), 1/12 (8.3%) and 1/4 (25.0%), 1/4 (25.0%), 0/4 (0.0%), respectively (*P* = 0.123). The grade in NISS of all 10 enrolled cases were listed in Table 1. The relationship between NISS and long-term ciliary process dysfunction secondary to ASI was depicted in Fig. 3.

### The relationship between 3 long-term ciliary process dysfunctions secondary to ASI

After 2 years of follow-ups, most PB (4/5) occurred 6 months after injury, but one occurred 1 year after injury (Case No. 18). Most hypotony and CHS (3/5) occurred within one month after injury, but two occurred at 6-month follow-up (Case No. 16,20). All cases (5/5) presenting with hypotony (Case No. 1,20,22) and CHS (Case No. 13,16) at 1-year follow-up remained stable during subsequent follow-up period. Two years of follow-up records of IOP in 6 patients presenting with CHS and hypotony within 12 months after injury were listed in Table 2.

**Table 2:** Two years of follow-up records of IOP in 6 patients presenting with CHS and hypotony at 6-month follow-up

Case No	Long-term complications at 2-year follow-up	IOP/△IOP (mmHg)			
		1-month	6-month	12-month	24-month
1	Hypotony	5.0/8.0	5.2/8.3	-	-
13	CHS	13.5/6.7	14.3/6.8	13.2/7.1	-
16	CHS	14.5/4.6	13.8/5.3	15.1/5.7	11.8/5.8
18	Hypotony	7.2/6.8	7.5/7.0	PB	
20	Hypotony	9.7/4.4	6.8/6.8	6.8/8.1	6.7/8.8
22	Hypotony	7.8/4.1	7.9/4.2	7.8/4.6	-

IOP means IOP of the affected eye at 1, 6, 12, 24 months of follow-up

△IOP means IOP of the contralateral healthy eye minus IOP of the affected eye at 1, 6, 12, 24 months of follow-up

*CHS* Ciliary hyposecretion, *IOP* Intraocular pressure, *PB* Phthisis bulbi

From a dynamic point of view, five of 8 cases (ASI grade 2, Case No. 1,2,3,4,6,18,20,22) (62.5%) presented with hypotony within 6 months after injury developed PB (ASI grade 3, Case No. 2,3,4,6,18) at 1-year follow-up, with the other 3 remaining unchanged (ASI grade 2, Case No. 1,20,22). Two patients presented with CHS (ASI grade 1, Case No. 13,16) within 6 months after injury neither worsened nor improved during subsequent follow-up. In addition, 3 other patients (ASI grade 0, Case No. 10,11,17) presented with ophthalmoplegia and mASI, but without any ciliary function damage, recovered well. A higher ASI grade from 0–3 within 6 months after injury predicted greater likelihood of subsequent PB at 2-year follow-up (0.0% vs. 0.0% vs. 62.5% vs. 100%). Main differential clinical features of the 4 grades of ASI were listed in Table 3.

**Table 3:** Main differential clinical features of the 4 grades of ASI

Grades	IOP of the contralateral healthy eye minus IOP of the affected eye > 5 mmHg	IOP of the affected eye < 8 mmHg	Positive Tyndall effect	Globe size of the affected eye / globe size before injury < 80%
0	**-**	**-**	**+/-**	**-**
1	** + **	+/-	**+/-**	**-**
2	**usually**	** + **	** + **	**-**
3	** + **	** + **	**usually**	** + **

+ means with the above condition;—means without the above condition; +/- means with or without the above condition; usually: usually with the above condition

*ASI* Anterior segment ischemia, *IOP* Intraocular pressure

### Analysis of risk factors for secretion dysfunction of ciliary process following HA filler injection

After 2 years of follow-ups, all of the 10 study group patients presented with ASI within 1 month and at 1-month follow-up after injury; while 2 and 0 out of 10 control group cases exhibited ASI at the above two follow-ups (100% vs. 20%, *P* = 0.001; 100% vs. 0%, *P* < 0.001, respectively). Six and eight patients in study group presented with ophthalmoplegia at 1-month follow-up and hypotony within 6 months; while 0 and 0 case in control group exhibited both clinical features (60% vs. 0%, *P* = 0.011; 80% vs. 0%, *P* = 0.001, respectively). Of particular concern is the fact that one case (Case No. 11) divided into NISS grade 1 without PB also developed ophthalmoplegia. Demographic and clinical features of the patients enrolled in the study and control groups were depicted in Table 4.

**Table 4:** Demographic and clinical features of the patients enrolled in the study and control groups

Parameters	All enrolled eyes (*n* = 20)	Study group (*n* = 10)	Control group (*n* = 10)	*p* value
Demographic				
Sex (male/female)	1/19	1/9	0/10	1.000
Age (years)	31.95 ± 7.84	33.00 ± 8.37	30.90 ± 7.58	0.564
Eye (right/left)	8/12	5/5	3/7	0.650
Clinical features				
Ophthalmoplegia within 24 h	8(40)	6(60)	2(20)	0.170
Ophthalmoplegia at 1-month follow-up	6(30)	6(60)	0(0)	0.011
ASI within 1 month	12(60)	10(100)	2(20)	0.001
ASI at 1-month follow-up	10(50)	10(100)	0(0)	< 0.001
NLP at 1-month follow-up	14(70)	9(90)	5 (50)	0.141
Iris atrophy of 360°circumference within 6 months	4(20)	4(40)	0(0)	0.087
Hypotony within 6 months	8(40)	8(80)	0(0)	0.001
NISS Grade 3	11(55)	7(70)	4(40)	0.370
NISS Grade 3–4	14(70)	9(90)	5(50)	0.141

*ASI* Anterior segment ischemia, *NISS* New injury severity scale, *PB* Phthisis bulbi

Data were presented as n. (%) or mean ± SD, unless otherwise indicated

### A novel predictive model for PB

Even in the total of 22 cases suffering from ocular/orbital complications following cosmetic filler injection, iris atrophy of 360°circumference within 6 months, hypotony with 6 months, ophthalmoplegia at 1-month follow-up and ASI at 1-month follow-up were individual clinical features that were better predictors for subsequent PB. Their AUCs for predicting subsequent PB were 0.90, 0.91, 0.88 and 0.85, respectively. Sensitivity, specificity and the AUC for the 7 scenarios (namely, scenarios A, B, C, D, E, F and G) were listed in Table 5. Obviously, the best scenario for predicting subsequent PB was scenario E (ophthalmoplegia at 1-month follow-up and hypotony with 6 months). Sensitivity, specificity and the AUC of scenario E was 100%, 100% and 1.00, respectively.

**Table 5:** Sensitivity, specificity and area under the receiver operating characteristic curve for each scenario

Scenarios	Features	Sensitivity	Specificity	1-Specificity	AUC	*p* value
A	Iris atrophy of 360°circumference within 6 months	4/5(0.80)	17/17(1.00)	0.00	0.90	0.008
B	Hypotony within 6 months	5/5(1.00)	14/17(0.82)	0.18	0.91	0.006
C	Ophthalmoplegia at 1-month follow-up	5/5(1.00)	13/17(0.76)	0.24	0.88	0.011
D	ASI at 1-month follow-up	5/5(1.00)	12/17(0.71)	0.29	0.85	0.019
E	B and C	5/5(1.00)	17/17(1.00)	0.00	1.00	0.001
F	B and D	5/5(1.00)	14/17(0.82)	0.18	0.91	0.006
G	C and D	5/5(1.00)	16/17(0.94)	0.06	0.97	0.002

*ASI* Anterior segment ischemia, *AUC* Area under the receiver operating characteristic curve

## Discussion

The cosmetic fillers used in all the 20 enrolled cases were HA gels, which is consistent with reports in the literature that the application of HA was more common than autologous fat [16]. Of the 20 cases, 5 ones developed PB. However, among the 10 cases of similar ocular complications caused by autologous fat reported in the literature [17–24], only 1 patient [24] who died showed PB. It can be seen that the possibility of PB caused by HA embolism (5/20) was far greater than that of autologous fat (1/10). We speculate that the possible reason is that HA microspheres are much smaller than autologous fat particles [16], so they are more likely to block the terminal branches of the ophthalmic artery (OA), including the ASC, resulting in ASI. The obstruction of the ASC, ranging from mild to severe, represents different degrees of ASI, resulting in CHS, hypotony, and PB, respectively (Fig. 2).

Regarding the definition of hypotony, although the World Glaucoma Association recommended a definition of less than 6.5 mmHg [9], the ocular trauma research literature often defined it as less than 8 mmHg [7]. In the cases of ASC embolization we observed, the IOP below 8 mmHg often showed variety degrees of ASI, and the mildest positive sign was Tyndall effect, so we adopted the definition in the ocular trauma study. The sASI caused by HA mainly occurred in NISS grades 3 and 4, which were characterized by immediate NLP. Surprisingly, the ASC was generally not affected before central retinal artery occlusion (CRAO) occurred. The possible reason is that the central retinal artery (CRA) is a terminal vessel without collateral circulation, and the diameter of the vessel is small, usually less than 0.2mm [25]; while the ASC usually consists of 7 anterior ciliary arteries (ACAs) and 2 long posterior ciliary arteries (LPCAs), which has abundant anastomoses with the conjunctival and ciliary arteries [10, 11]. Thus, CRAO requires only a small amount of HA microspheres to occur; clinically significant ASI requires a large number of HA microspheres to retrograde into the ASC. This theoretically explained why sASI usually occurred in NISS grades 3 and 4 cases with immediate NLP, with NISS grades 3 and 4 indicating retrograde entry of large amounts of HA microspheres into the OA, secondary occurrence of CRAO, and consequent greater likelihood of sASI.

According to our observation, PB caused by HA embolism mostly (4/5) occurred about half a year after the injury (Table 1). The interval from HA embolism to PB was similar to 0.9 years of infectious endophthalmitis, but significantly shorter than 1.4 and 2.9 years of ocular trauma and uveitis, respectively [26]. HA embolism-induced hypotony and CHS at 2-year follow-up mostly (3/5) occurred within 1 month after injury, and this pathological state usually remained unchanged for subsequent 2 years. A various recti recession experiment with monkey eyes [10] also found that the low IOP usually occurred within 1 month after the surgery. The difference was that the low IOP caused by the recti recession surgery will usually recover spontaneously with the stabilization of the ASC, and generally no PB will occur. It can be seen that the consequences of ASI caused by extensive embolization of ASC by HA were much more serious than those of recti recession surgery.

With regard to the pathophysiological mechanism of PB, we speculated that a large number of HA microspheres retrograde through the OA first entered the extraocular muscular artery (EMA), ACA, LPCA, and CRA, leading to the embolism of these arteries. The ACA is derived from the EMA, which together with the LPCA and the scleral perforating branches of the conjunctival artery forms the ASC [10]. Subsequently, The CRAO resulted in immediate NLP; occlusion of the EMA resulted in ophthalmoplegia; extensive occlusion of the extraocular muscular-anterior ciliary artery (EMACA) and the LPCA together resulted in severe hypoperfusion of the ASC, ultimately leading to sASI. The co-occurrence of 360-degree circumferential iris atrophy and hypotony within 6 months after extensive occlusion was the most important features of sASI. As a result, sASI meant a very high probability of PB at 6-month follow-up.

In this study, ASI was graded mainly based on the degrees of ciliary process dysfunction, rather than iris fluorescein angiography and anterior segment abnormalities (such as segmental iris atrophy, pupil irregularity, uveitis) adopted in previous article [27]. Ciliary process dysfunction was mainly reflected in the changes of IOP, so the grading system we proposed mainly focused on the fluctuations of IOP and whether it was combined with Tyndall effect reflecting the disruption of the blood-ocular barrier. This grading system based on dysfunction had several advantages. First of all, it was convenient, easy to master, and suitable for widespread promotion. Secondly, the grading system did not require special equipment, so it was non-invasive and suitable for assessment and dynamic observation at any time. Last but not least, it could accurately reflect the severity of ASI and the possibility of subsequent PB.

Of course, there are still some deficiencies that deserve attention in this study. First, due to the rarity of such catastrophic consequences, only 10 long-term ciliary process dysfunctions secondary to ASI occurred even after 4 years of enrollment. Therefore, the scientific nature of the grading system still needs to be verified by a large number of clinical practices. In addition, this is a retrospective study, and some patients lack IOP follow-up data at certain time points; some patients lack detailed anterior segment evaluation, such as ultrasound biomicroscopy and gonioscopy, etc. Even so, we still believe that the grading system of ASI mentioned in this paper has a more accurate role in evaluating the long-term ciliary function damage caused by rectus muscles regression surgery or occlusion of ASC, and it is worthy of clinical reference.

## Conclusions

The new grading system for ASI and novel predictive model for PB we proposed could predict the long-term prognosis and probability of subsequent PB due to ASI following HA injection through several dynamic assessments within 6 months. Therefore, we suggested that patients with ophthalmoplegia at 1-month follow-up and persistent hypotony within 6 months should be fully informed of the extremely high possibility of subsequent PB.

## Data Availability

The data that support the findings of this study are available on request from the corresponding author, [YH], upon reasonable request.

## Abbreviations

ACA: Anterior ciliary artery
ASC: Anterior segment circulation
ASI: Anterior segment ischemia
AUC: Area under the receiver operating characteristic curve
CHS: Ciliary hyposecretion
CRA: Central retinal artery
CRAO: Central retinal artery occlusion
EMA: Extraocular muscular artery
EMACA: Extraocular muscular-anterior ciliary artery
HA: Hyaluronic acid
IOP: Intraocular pressure
LPCA: Long posterior ciliary artery
NISS: New injury severity scale
NLP: No light perception
OA: Ophthalmic artery
OAO: Ophthalmic artery occlusion
PB: Phthisis bulbi
RAPD: Relative afferent pupillary defect
